# Detection and Characterization of Antibiotic-Resistant Bacteria Using Surface-Enhanced Raman Spectroscopy

**DOI:** 10.3390/nano8100762

**Published:** 2018-09-26

**Authors:** Kaidi Wang, Shenmiao Li, Marlen Petersen, Shuo Wang, Xiaonan Lu

**Affiliations:** 1 Food, Nutrition and Health Program, Faculty of Land and Food Systems, The University of British Columbia, Vancouver, BC V6T1Z4, Canada; kaidi.wang@ubc.ca (K.W.); shenmiao.ivy.li@gmail.com (S.L.); Marlen.Petersen@web.de (M.P.); 2 Tianjin Key Laboratory of Food Science and Health, School of Medicine, Nankai University, Tianjin 300371, China; wangshuo@nankai.edu.cn

**Keywords:** SERS, chemometrics, resistance, biosensing, rapid detection

## Abstract

This mini-review summarizes the most recent progress concerning the use of surface-enhanced Raman spectroscopy (SERS) for the detection and characterization of antibiotic-resistant bacteria. We first discussed the design and synthesis of various types of nanomaterials that can be used as the SERS-active substrates for biosensing trace levels of antibiotic-resistant bacteria. We then reviewed the tandem-SERS strategy of integrating a separation element/platform with SERS sensing to achieve the detection of antibiotic-resistant bacteria in the environmental, agri-food, and clinical samples. Finally, we demonstrated the application of using SERS to investigate bacterial antibiotic resistance and susceptibility as well as the working mechanism of antibiotics based on spectral fingerprinting of the whole cells.

## 1. Introduction

Detection of pathogenic and spoilage bacteria is still a major concern to clinical, agri-food, and environmental agencies and laboratories [1]. The leading challenge is the detection speed [1]. Since the contamination level of bacteria may be relatively low and the sample matrices can significantly influence accurate and reproducible detection, extensive sample preparation steps are always required to separate the targeted bacteria from the sample matrices along with pre-enrichment [2,3]. Because the detection includes all the times starting from obtaining the samples to the signal readout, both separation and bacterial enrichment account for most of the times for bacterial detection rather than the final real detection using an instrument or a sensor [4]. For example, the conventional plating assay will take several days to confirm the growth of the targeted bacterial colony [5]. In comparison, molecular-based detection methods, such as polymerase chain reaction (PCR), requires relatively less time than the plating assay but still cannot fully avoid separation and bacterial pre-enrichment [6]. Recently, matrix assisted laser desorption ionization time-of-flight (MALDI-TOF) spectrometry has attracted considerable interest for the rapid identification of pathogens by profiling bacterial proteins from the whole cells [7]. However, this method is not suitable for characterizing a mixed sample [8] and still requires the priori cultivation and sample preparation procedure [9]. An alternative method is surface-enhanced Raman spectroscopy (SERS), an advanced Raman spectroscopic technique that enhances the vibrational modes of molecules adsorbed on or in the vicinity to the surface of metal nanoparticles. SERS provides rapid, ultra-sensitive and accurate detection with minimum requirement for sample handling and preparation.

Antibiotic resistance of pathogenic bacteria is still a leading concern to clinics as well as agri-food and veterinary medicine [10]. The key battle is to perform an accurate diagnosis of the pattern of bacterial antibiotic resistance in an early manner. Otherwise, only the broad-spectrum antibiotics can be used to treat this type of bacterial infections [11]. As aforementioned, the conventional microbiological testing, such as the determination of minimum inhibitory concentration (MIC) using the broth microdilution method, is highly time-consuming. Besides, PCR-based testing of the targeted antibiotic-resistant genes requires highly trained personnel and has a potential risk of cross contamination [12,13]. Another major limitation of this approach is that the presence of the resistance genes may not necessarily confer to the clinically relevant phenotypic resistance of bacteria [14]. Microarray offers the ability to detect a broad range of resistance genes present in the bacterial isolates with high sensitivity and specificity. However, similar to the PCR-based method, results obtained from microarrays may not always correlate to the phenotypic resistance [14]. Although MALDI-TOF mass spectroscopy can potentially differentiate the resistant and susceptible isolates based on the spectral features [7], it requires additional chemicals as the matrix for the performance of MALDI [14]. Alternative technology that can detect and characterize bacterial antibiotic resistance is therefore highly required. SERS is a powerful biochemical fingerprinting technique as it can accurately reflect the macromolecular profiles and changes that occur within the bacterial cells due to the action of the antibiotics [15].

In this mini-review paper, we will evaluate the use of SERS coupled with chemometrics as a tool to detect the trace level of antibiotic-resistant bacteria and characterize the mechanism of bacterial antibiotic resistance in an ultra-fast manner. The recent progress in this research area will be summarized and discussed mainly focusing on the following three perspectives: (1) the nanomaterials that can be used as the SERS substrates for sensing a low concentration of bacterial cells; (2) tandem-SERS technology to detect antibiotic-resistant bacteria in a sample matrix; and (3) characterizing the mechanism of bacterial antibiotic resistance and susceptibility using SERS and chemometrics.

## 2. Surface-Enhanced Raman Spectroscopy (SERS) for Sensing Trace Level of Bacteria

### 2.1. Mechanism of SERS

**Figure 1:**
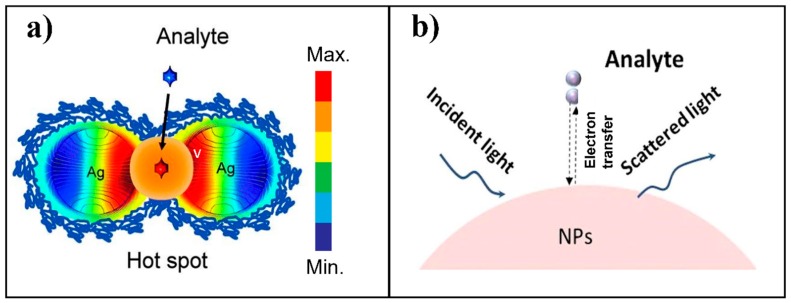
Two mechanisms contributed to surface-enhanced Raman spectroscopy (SERS). (**a**) Electromagnetic enhancement of SERS-active silver nanoparticles. SERS “hot-spot” is generated in the gap between two close nanoparticles. (**b**) Chemical enhancement resulting from electron transfer between analytes and the surface of nanoparticles. Reproduced with permission [31]. Copyright Royal Society of Chemistry, 2014. Reproduced with permission [32]. Copyright Elsevier B.V., 2017.

SERS is a derivative of Raman spectroscopy with the aid of nanomaterials. Numerous research studies have been conducted during the past four decades about using SERS for trace detection of the targeted analytes [16,17,18,19,20,21,22,23]. Different from the conventional Raman spectroscopic technology, SERS signal can be significantly enhanced due to both electromagnetic enhancement and chemical enhancement, with the former being the dominant contributor [24]. Electromagnetic enhancement is generated from the localized surface plasmon resonance (LSPR) in the vicinity of the nanostructured surface of noble metals, such as silver and gold [25,26]. Highly localized regions of amplified electromagnetic fields caused by LSPR are called “hot spots”, which usually occurs in the gaps, crevices, or sharp vertices of supporting plasmonic materials (Figure 1a). In comparison, chemical enhancement is due to the electron transfer between the analyte molecule and the surface of the nanostructure when the energy of the incident light matches the electron transfer energy (Figure 1b) [27]. This will lead to the change of molecular polarization and subsequently enhance the Raman signal approximately 100 times. Theoretically, total SERS enhancement factors may approach to ~10^14^ depending on the nanomaterials used. For additional details, the authors are encouraged to refer to serial publications from the Van Duyne research group [27,28,29,30].

### 2.2. SERS-Active Substrates for Bacterial Detection

Because SERS can reach to single molecule detection, it has been widely applied for the detection of various analytes in an ultra-fast manner (e.g., a few seconds to less than a minute). In general, the reproducibility of the SERS signal is getting worse along with the increase of the size of the analyte [33]. For example, it is extremely challenging to harvest a reproducible SERS signal for a bacterial cell than that of a small chemical molecule, such as antibiotics and pesticides [34]. Although successful discrimination of bacteria by using SERS was reported by different research groups [15,35,36], the real world application is still extremely challenging, such as the low concentration of the targeted bacteria in the sample and a relatively large amount of interference sample components. Therefore, researchers have been developing various types of SERS-active substrates to enhance the signal intensity as well as generate more reproducible SERS signals for different biological samples, such as bacteria and viruses. Both “top-down” and “bottom-up” methods have been used for the synthesis of SERS-active substrates [37]. For the “top-down” method, large multi-dimensional materials are reduced to ideal nanoscale structures using direct fabrication process [38]. In comparison, the “bottom-up” method refers to the development of complex nanoscale structures from simple molecules or atoms [39].

#### 2.2.1. Direct SERS

Generally, SERS-active nanostructures are composed of two types of substrates: solid surface-based substrates and colloidal substrates. The solid surface-based substrates can accurately control the formation of “hot spots”. Once the bacteria cells are closed to the “hot-spot” on the surface of the solid substrate, a significant SERS effect will be achieved. For example, a recent study presented a label-free SERS-based method to detect and identify *Salmonella enterica* and *Escherichia coli* adsorbed on the silver dendrites [40]. Since the nanoparticles were already closely aligned on the stem and branches, “hot spots” could be generated without any aggregation process. This also contributed to producing uniform and homogenous sample spots after drying, which eliminated the spot-to-spot variation of the collected SERS signals. SERS spectra collected using the silver dendrites were consistent and robust enough for the detection and identification of bacteria with a limit of detection (LOD) as low as 10^4^ colony-forming unit (CFU) per mL. Besides, porous anodic aluminum oxide (AAO) has been widely used as the substrate for the synthesis of functional nanostructures by coating a thin layer of gold or silver to develop a nanostructured noble metal substrate to enhance SERS signal intensity [41]. Ji and co-authors reported a three-dimensional nanostructure fabricated by depositing silver NPs into AAO templates using a simple electrochemical deposition method [42], demonstrating well-ordered micro/nanostructures when it was characterized by field emission scanning electron microscopy. The homogeneity of SERS substrates is the key to the reproducibility of SERS spectra and even minor variation in the surface morphology can result in significant changes in the enhancement. Due to the well-organized structure of decorated AAO membranes, the distribution of “hot-spots” is uniform, which can eventually improve the SERS spectral reproducibility [43].

In addition, various colloid systems of gold or silver have been synthesized as the liquid format of SERS substrates for the detection of bacterial cells [44]. A more uniform distribution of noble metal nanoparticles on the surface of bacterial cells can be achieved to improve the SERS spectral reproducibility compared to that by using the solid SERS substrates [45]. A SERS application employing a synthesis of silver nanocolloids coating on a bacterial cell wall can detect the live bacteria in drinking water down to 2.5 × 10^2^ CFU/mL [46]. Another study conducted by Chen and colleagues applied Ag colloids for the discrimination of *E. coli*, *Pseudomonas aeruginosa*, methicillin-resistant *Staphylococcus aureus* (MRSA) and *Listeria*. In situ synthesis of Ag nanoparticles and the addition of Triton X-100 significantly improved the sensitivity of SERS detection [47]. A simple method of preparing SERS substrates was described by filtering Ag or Au colloidal particles onto a ceramic filter, onto which the bacterial suspensions were then filtered [48]. This method allowed the homogeneous distribution of bacteria on the surface of the substrate, which increased the sensitivity of SERS detection. A microfluidic “lab-on-a-chip” platform can be used to further improve the reproducibility of SERS signal by mixing the silver/gold nanocolloids with bacterial cells in a controlled fluidic manner with limited precipitation of individual nanoparticles on the substrate, in which case the channel in the microfluidic device could avoid spectral interference and enhance the sensitivity of bacterial detection [49]. SERS-microfluidic systems have been used to classify multiple foodborne pathogens using chemometrics and quantify single pathogenic bacterial cells. For example, Mungroo and others successfully distinguished eight foodborne pathogenic bacterial species using microfluidic-integrated SERS substrate and chemometrics, including principal component analysis (PCA) and linear discriminant analysis (LDA) [50]. A SERS-based microfluidic system was developed for the discrimination of *E. coli* strains with the spectral recording time reduced to 1 s [51]. Ag nanoparticles were injected into the bacterial suspension to facilitate the aggregation of nanocolloids on the bacterial cells. Besides, a SERS substrate composed of 3D Ag@ZnO nanostructures was also integrated into a microfluidic device for SERS fingerprinting detection of a single living cell [52]. Colloidal substrate seems to be more popular due to its simple and cost-effective fabrication, but solid surface-based substrates are more favorable for the detection of water-insoluble substances [53]. A variety of SERS nanomaterials used for bacterial biosensing have been summarized in Table 1.

SERS has been widely applied for the differentiation of antibiotic-resistant strain and antibiotic-sensitive strains possibly due to the variation in the biochemical compositions of bacterial cell membrane and cell wall. In a recent study, Li and others reported that surface-enhanced resonance Raman spectroscopy (SERRS) could achieve almost a 100% accuracy for the differentiation between carbapenem-resistant *E. coli* and carbapenem-sensitive *E. coli* [82]. Lu and coauthors developed a microfluidic SERS platform for a successful high-throughput screening and differentiation between MRSA and methicillin-sensitive *Staphylococcus aureus* (MSSA). In addition, the SERS characterization of bacterial phenotypic profiles had a good correlation to the multilocus sequence typing as well as antibiotic characterization using PCR, demonstrating the possibility of applying SERS as the alternative to detect antibiotic resistance and track the outbreak of pathogenic bacteria [54]. In another study, Mühlig and coauthors applied a similar SERS microfluidic chip for the differentiation of various species of mycobacteria, including both nontuberculous mycobacteria and *Mycobacterium tuberculosis* complex [55].

#### 2.2.2. Indirect SERS

**Figure 2:**
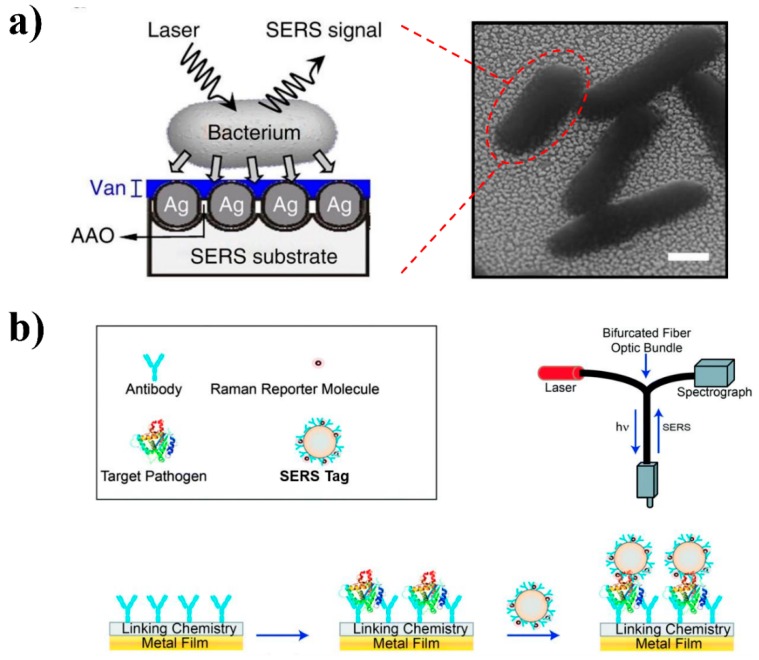
Representative “direct” (**a**) and “indirect” (**b**) SERS detection of bacteria. (**a**) Schematic diagram showing the SERS signal was directly collected from the bacterium on a vancomycin-coated Ag/AAO SERS-active substrate (left). Scanning electron microscope (SEM) image of bacteria on the substrate (scale bar, 500 nm) (right). (**b**) Schematic illustration of a sandwich-like indirect antibody-SERS detection. Key steps including: immobilization of antibody on the surface of metal substrate; capture of target bacteria by modified surface and labeling the target bacteria with SERS tag for detection. Reproduced with permission [83]. Copyright Springer Nature, 2011. Reproduced with permission [72]. Copyright Royal Society of Chemistry, 2011.

The aforementioned SERS substrates are related to “direct sensing” of the analyte (e.g., a bacterium) by using a laser with the wavenumbers of mainly 532, 633, and 785 nm [53]. In other words, the collected SERS spectral features are directly associated with the chemical compositions of the targeted bacteria (Figure 2a). In comparison, SERS tags have been designed and used for “indirect sensing” of the analyte(s) (Figure 2b).

**Figure 3:**
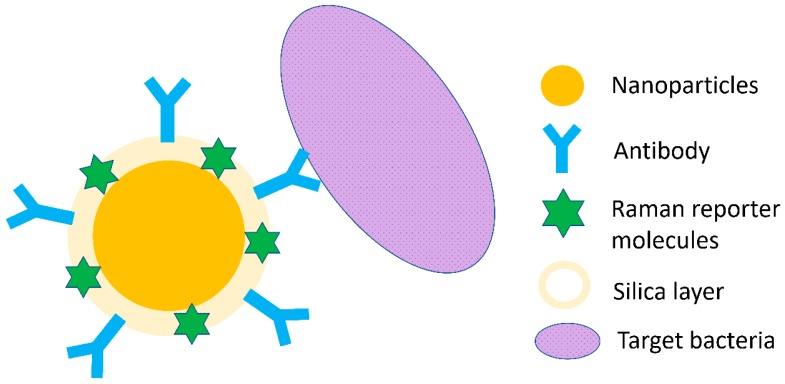
Schematic illustration of SERS tags.

The schematic illustration of the SERS tag is shown in Figure 3. Specifically, a SERS-active molecule, such as rhodamine 6G, will be used as the tag molecule for the synthesis of a gold/silver nanostructure [72]. By conjugating with a separation element, such as an antibody, aptamer, or a molecularly-imprinted polymer, a functional SERS tag will be developed. This SERS tag can specifically recognize and capture the targeted analyte (e.g., a bacterium) from a complicated sample matrix to achieve separation and possibly enrichment as well [32].

**Figure 4:**
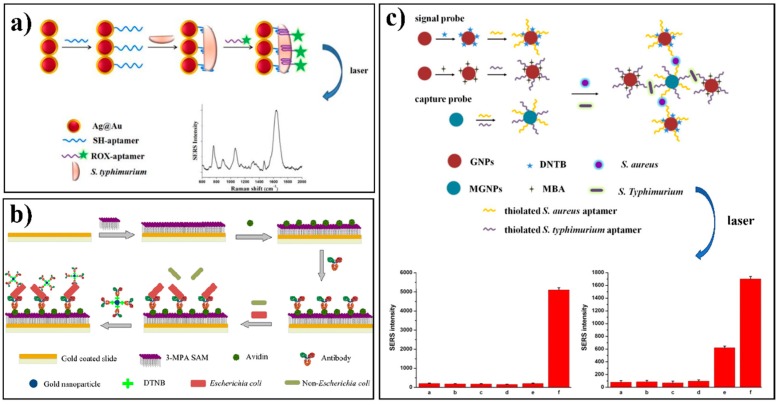
(**a**) Schematic illustration of aptamer-based SERS approach for the detection of *Salmonella* Typhimurium. Ag/Au core/shell nanoparticle was conjugated with a specific aptamer. The Raman reporter, X-rhodamine (ROX), was labeled on the same aptamer sequence. Nanoparticle-aptamer-target-aptamer-Raman reporter complexes enabled SERS detection. (**b**) Schematic illustration of the antibody-based sandwich-type SERS immunoassay for *Escherichia coli* enumeration. SERS tags were constructed by gold nanoparticles first coated with a Raman reporter molecule, 5,5′-dithiobis (2-nitrobenzoic acid) (DTNB), and subsequently with a corresponding antibody. (**c**) Multiplex detection of *Salmonella* Typhimurium and *Staphylococcus aureus* using aptamer-SERS immunoassay. Fe_3_O_4_ magnetic gold nanoparticles were labeled with unique Raman reporters and aptamers against *S. aureus* and *S*. Typhimurium and then employed into a sandwich-like assay. Reproduced with permission [73]. Copyright Elsevier B.V., 2015. Reproduced with permission [89]. Copyright Springer-Verlag, 2010. Reproduced with permission [76]. Copyright Elsevier B.V., 2015.

Most indirect approaches use a sandwich-like immunosorbent assay format, which is similar to enzyme-linked immunosorbent assay (ELISA) [68]. The schematic illustration in Figure 2b shows the basic steps for developing a representative sandwich-structured indirect antibody-SERS method. Firstly, capturing antibodies are immobilized on the surface of a metal substrate. The second step is to capture the targeted pathogen from the sample matrix using these immobilized antibodies. Finally, the SERS tag will be introduced to label the targeted pathogen for Raman signal collection. The availability of the collected SERS signal is derived from the SERS tag molecule, but can indirectly indicate the availability and the concentration of the targeted bacteria in the sample matrix. This indirect SERS-tag technology is extremely useful for the detection of bacteria in a complicated sample matrix, such as a food, because the aforementioned direct SERS detection can be significantly affected by the food sample matrix if the sample pre-treatment is not fully complete [37]. For example, Duan and co-authors reported an indirect SERS-based method for the quantification of *S.* Typhimurium in milk (Figure 4a) [73]. *S.* Typhimurium interacted with Fe_3_O_4_/Au core/shell nanoparticles functionalized with specific aptamers and Raman reporters in conjugation to the same aptamer to form a sandwich-like complex. A linear correlation for bacteria concentration of ~10–10^6^ CFU/mL and a low LOD of 15 CFU/mL were obtained in this study. *Vibrio parahaemolyticus* was successfully detected in shrimp and water samples using a similar approach [74]. The specific aptamer immobilized on the SiO_2_-core-Au-shell nanoparticles was used to selectively capture *V. parahaemolyticus*, leading to a LOD of 10 CFU/mL. In another study, silver nanoparticles functionalized with antibodies and Raman reporter to serve as the SERS tags were successfully applied for rapid detection of *E. coli* to a concentration as low as 10^2^ CFU/mL [63]. Although several publications demonstrated a good separation capability and spectral reproducibility by integrating silver/gold nanoparticles with magnetic materials [84,85,86], we still believe a functional SERS tag with separation element is more effective at the current stage. More precise control of the numbers and orientations of the molecules on the surfaces of the magnetic nanoparticles have to be achieved [84]. In addition, a few studies reported the development of functional SERS tags by integrating both separation elements and magnetic beads to achieve an even better separation, enrichment, and signal enhancement capability [62,70,80,87]. For example, an LOD of 35 CFU/mL and LOQ of 3.5 × 10^2^ CFU/mL for *E. coli* was reported using a combination of antibody-modified magnetic nanoparticles and gold nanorods labeled with the same antibodies in a sandwich-format detection strategy [62]. Besides, a recent study conducted by Kearns and colleagues reported a novel assay of using lectin-functionalized magnetic nanoparticles along with SERS-active nano-substrates functionalized with various antibodies to successfully capture and detect multiple antibiotic-resistant pathogens, including *Salmonella*, *E. coli* and MRSA at the single cell level in a simultaneous manner [88].

## 3. Tandem-SERS for Sensing Bacteria in a Sample Matrix

### 3.1. Tandem-SERS Methods

Tandem-SERS refers to conjugating the separation element to the SERS system that can achieve separation and detection simultaneously [90]. The aforementioned functional SERS tag with a separation element (e.g., antibody, aptamer, molecularly-imprinted polymer) is a classical tandem-SERS system. Due to the size of bacterial cells, a sandwich tandem-SERS structure is always developed [15,68] and the detailed illustration is shown in Figure 2b. Antibody is widely used as the recognition element due to its specificity to bacteria via a covalently-bound effect. An antibody conjugated with different SERS nanoprobes such as Ag@silica core-shell nanoparticles [71], popcorn-shaped Au nanoparticles [72], and single walled carbon nanotubes-Au nanoparticles [91] was used to detect normal *Salmonella* or multi-drug-resistant *Salmonella*. High correlation coefficients and LOD of 4 and 5 CFU mL^−1^ were obtained using an antibody-SERS employing AuNPs via a sandwich immunoassay for detecting and enumerating *E. coli* (Figure 4b) [89]. The results of testing bacteria in lake and tap water samples were highly consistent with that of the classical plating assay.

**Figure 5:**
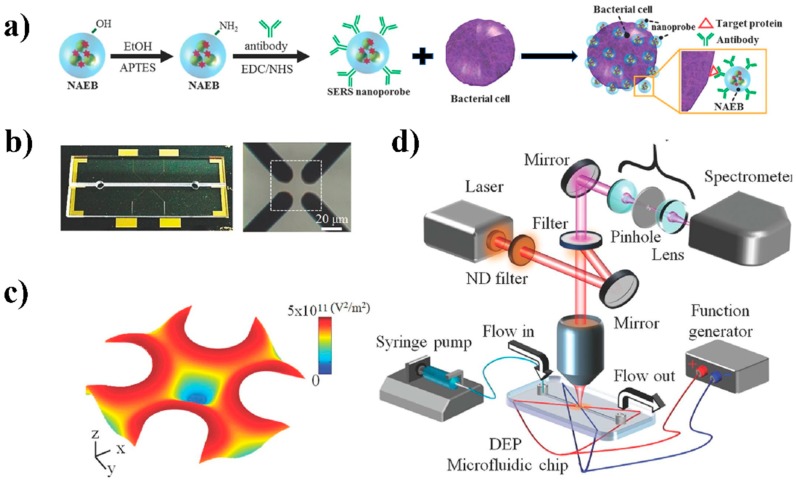
The integration of SERS nanoprobes and a microfluidic dielectrophoresis (DEP) device for rapid detection of single bacterium. (**a**) Schematic presentation of using antibody-conjugated nanoaggregate-embedded beads (NAEBs) as SERS nanoprobes for specific detection of bacteria. (**b**) Photograph of the microfluidic DEP device and close-up view of central capturing area with four the quadrupole electrodes. (**c**) The distribution of electric field of four microelectrodes in the microchannel. (**d**) Schematic illustration of the DEP-SERS configuration. Reproduced with permission [93]. Copyright WILEY-VCH Verlag GmbH & Co. KGaA, Weinheim, 2014.

Aptamer is another element that can be used and conjugated in tandem-SERS for the recognition, separation, and enrichment of specific bacterial pathogens. Aptamer-based SERS assay was able to monitor photothermal activity response of MRSA and multi-drug-resistant *Salmonella* DT104 through the change of Raman signal intensity of R6G [32]. Zhang and coauthors reported a simultaneous detection of *S*. Typhimurium and *S. aureus* using Au NPs-aptamer based SERS biosensor (Figure 4c). A high sensitivity with LOD of 35 and 15 CFU/mL for *S. aureus* and *S.* Typhimurium was achieved, respectively [76]. Another format of tandem-SERS was to include SERS sensing in a microfluidic device. A complicated design of the microfluidic device can realize the function of separation of bacterial cells from the sample matrix mainly [49]. Dielectrophoresis is an effective method for concentrating and trapping various types of nanoscale/microscale particles in a microfluidic device, including microorganisms [92]. It is also feasible to conjugate the aforementioned separation elements, such as aptamer, onto the microchannels to form a more comprehensive and effective tandem-SERS platform for simultaneous separation and detection [67]. Lin and co-authors developed a fast single-step SERS detection of *E. coli* O157:H7 at single cell level with speciation capability to sub-species. This was achieved by a multiplexing dual recognition SERS platform that combined specific antibody conjugated SERS tags with a microfluidic dielectrophoresis (Figure 5) [93].

### 3.2. Tandem-SERS Integrated with Multiple Capabilities

Another major application advantage of such a tandem-SERS platform is to enrich the bacterial cells and subsequently improve the detection sensitivity. Although SERS can theoretically detect a single molecule/cell, its real world application can only detect ~10^3^ CFU/mL of bacteria, mainly due to the interference from the sample matrix components [94]. Therefore, a relatively large amount of samples therefore is required for the production of a meaningful SERS signal readout. In a recent study reported by Zhang and others, the SERS-active substrate composed of gold nanoparticles was integrated into the microfluidic device for rapid concentration and detection of *S. aureus* in liquid samples [95]. The SERS signal intensity of *S. aureus* after concentration in this device was over 100-fold compared to the signal obtained from the raw sample, leading to a LOD of 2 × 10^2^–2 × 10^4^ CFU/mL. Hou and colleagues demonstrated a microfluidic system based on a discharge driven vortex technique to concentrate a bacterial suspension of *E. coli* F-amp and *Bacillus subtilis* for SERS detection. The combination of SERS and microfluidic with immunoassay techniques was able to selectively capture the targeted bacterial cells [96]. A SERS-based sandwich immunoassay employing antibody-coated magnetic nanoparticles for *E. coli* enumeration was also reported [97]. The authors accomplished a LOD of 8 CFU/mL by combining bacterial separation with SERS detection using specific SERS labels.

Combination of SERS platform and a filter (e.g., polymer fiber) has been recently used for the identification and detection of bacteria from clinical and environmental samples. For instance, Lin and others demonstrated a filter-like SERS substrate prepared with AuNPs embedded in mesoporous silica for the detection of *Staphylococcus aureus* from the aqueous samples [98]. The targeted cells could be concentrated on the filter-like substrates within a few seconds. Strong SERS signals with good bacterial discrimination were obtained without any need for pre-labeling, and the reproducibility was also significantly improved. More recently, Kamińska and colleagues presented a new label-free tandem-SERS platform for rapid detection of *Neisseria meningitidis* [99]. This bacterium is a Gram-negative diplococcus and one of the three major bacteria that cause acute bacterial meningitis. The applied SERS substrate was based on Si/ZnO layers and electrospun polymer mats covered with a thin layer of sputtered gold. A wide range of pore sizes makes the polymer mat an excellent material to filter bacteria from fluids and then immobilize them onto the SERS nanostructures for the collection of Raman signals, enabling the detection of single bacterial cells of *N. meningitidis* present in cerebrospinal fluid samples. A similar approach was developed to detect bacteria from blood plasma [100]. Covering the forcespun polymer mat with Au/Ag alloy turns it into a SERS-active platform, which can be used as a filter to separate the microorganisms from fluids and immobilize them on the surface of the mat during the measurement. *S. aureus*, *Pseudomonas aeruginosa*, and *S.* Typhimurium were successfully detected and identified from blood plasma using the developed platforms. These SERS-active nanostructures based on polymer mats provide the possibility for simultaneous filtration, immobilization, and enhancement of Raman signals in a few seconds, demonstrating a simple and low-cost method to analyze bacterial suspensions in biological fluids with SERS.

In addition, the tandem-SERS platform can achieve multiplex detection of bacteria by integrating several different elements into a single system. By using a systematic evolution of ligands by exponential enrichment (SELEX), different aptamers can be synthesized and each one targets one species of bacteria. By conjugating the aptamers onto a substrate, such as the microchannel in a microfluidic device, the mixture of bacterial cocktails can be individually captured by each aptamer that eventually achieve multiplex detection in a simultaneous manner. For example, *S.* Typhimurium and *S. aureus* were simultaneously identified using different aptamers in a sandwich-type tandem-SERS detection within 3 h [76]. Sandeep and co-workers proposed another simple and robust cross-platform approach using different nanoparticles functionalized with specific capturing ligands and Raman reporter molecules. This multiplex detection platform was applied for simultaneous detection of three different pathogens and offered an LOD ranging between 10^2^ and 10^3^ CFU/mL with a total detection time less than 45 min [64].

### 3.3. “Two-Step” and “One-Step” SERS

**Figure 6:**
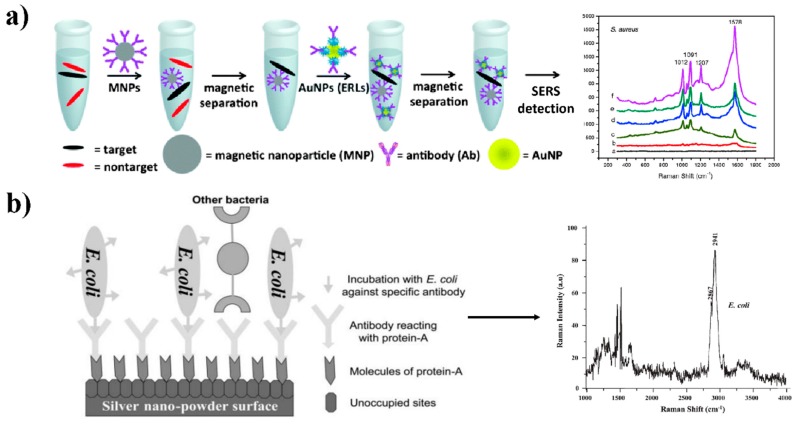
Representative “two-step” (**a**) and “one-step” (**b**) tandem-SERS sensing methods. (**a**) Tandem-SERS platform composed of the magnetic-based separation and SERS detection for multiple pathogens in food matrices. Pathogens were first captured with silica-coated magnetic probes, and then pathogen specific SERS probes (gold nanoparticles integrated with a Raman reporter and corresponding antibodies) were deployed to complete the following detection. (**b**) Schematic diagram for SERS-based detection of *E. coli* using silver nanoparticles conjugated with antibodies. Reproduced with permission [70]. Copyright Springer-Verlag, 2010. Reproduced with permission [102]. Copyright Royal Society of Chemistry, 2007.

In comparison to the aforementioned concepts of “direct sensing” and “indirect sensing”, “two-step sensing” and “one-step sensing” is another pair of the terminologies that are related to tandem-SERS platform. Once the separation and SERS detection are separate, it refers to “two-step” sensing. An intriguing “two-step” SERS approach based on a sandwich assay for the separation and detection of multiple pathogens in food samples was demonstrated by Wang and co-authors [70]. Figure 6a depicted the key steps of the process. The targeted pathogens in a food matrix were first captured and separated using silica-coated magnetic nanoparticles functionalized with the corresponding antibodies. Then, AuNPs integrated with a Raman reporter and surface-modified antibodies specific to the pathogen were used to complete the SERS detection. This platform achieved a LOD of 10^3^ CFU/mL for multiplex detection of *S*. Typhimurium and *S. aureus* in spinach wash water and peanut butter. “One step” sensing indicates that the separation and detection can occur simultaneously. Once “one-step” sensing is applied, a critical parameter is to ensure that the distance of the separation element is within 10 nm from the SERS-active substrate [90]; otherwise, the SERS effect will be tremendously reduced [101]. Naja and coauthors presented a “one-step” sensing of bacteria using silver nanoparticles functionalized with antibodies (Figure 6b). When the model bacteria attached to the corresponding antibodies absorbed on the protein-A-modified silver nanoparticles, the distance between the bacterium and the nanoparticle surface was 8 nm, thus the SERS signal of the bacterial cell wall would be generated and detected [102]. Further, “one-step” tandem-SERS sensing requires a relatively more complete clean-up of the sample matrices than that of the “two-step” tandem-SERS sensing method [90].

## 4. Elucidating Antibiotic Resistant Mechanism of Bacteria Using SERS and Chemometrics

Besides the detection of antibiotic resistant bacteria either in a simple matrix or a complicated environmental, agri-food or clinical sample matrix, another major research direction of using SERS is to study the working mode and mechanism of antibiotics to inactivate bacteria. Bacterial cells can develop various strategies to resist to the antibiotic treatment as the pinnacle of evolution. Although new antibiotic resistance has been continuously emerging and spreading globally, bacteria use is one of two leading genetic strategies to deal with antibiotic treatment, namely mutation in genes associated with the action of antibiotic compounds and the acquisition of external DNA for the resistance determinants through horizontal gene transfer [103]. These genetic variations will lead to the change in biochemical composition of the bacterial cells. For example, three different biochemical routes can arise, fluoroquinolone resistance, including over-expression of efflux pumps to extrude the antibiotics from the bacterial cells, mutations in genes encoding DNA gyrase and topoisomerase, and generating specific proteins to protect the targeted site of fluoroquinolone [104,105].

### 4.1. Characterization of Antibiotic Resistance of Bacteria Using SERS

As a three-dimensional complex surrounding the bacterial cells, peptidoglycan is the major component of the bacterial cell wall [106]. Since a relatively large amount of antibiotics is designed to target the bacterial cell wall, the biochemical compositions of the bacterial cell wall are expected to change along with the treatment of these antibiotics. Because SERS can record the macromolecular fingerprints of the bacterial cell membrane and cell wall, it can be applied to determine the effectiveness of antibiotic treatment as well as the antibiotic resistance patterns of the bacterial cells [15]. Although conventional Raman spectroscopy has been widely applied to profile the phenotypic response of bacteria to the antibiotic treatment, it requires a high concentration of bacterial culture for the collection of Raman signal [107]. Therefore, a relatively long time for bacterial cultivation and enrichment is necessary. By applying SERS for characterization, the antibiotic-resistant pattern of a single bacterial cell can be achieved. In addition, it will be critical to study the variations in responses among individual cells to the antibiotic treatment.

Antibiotic susceptibility testing (AST) is used to evaluate the effectiveness of antibiotic treatment against the pathogen infections. SERS-based AST could reduce the time by avoiding the need for overnight culture in MIC determination through the conventional AST methods. Liu and coauthors used an SERS-active substrate made of AgNPs imbedded in AAO to determine the antibiotic sensitivity of *E. coli* and *S. aureus* at the single-bacterium level [108]. Antibiotic-sensitive bacteria could be differentiated from antibiotic-resistant ones within 1 h after antibiotic exposure by monitoring the characteristic changes in SERS spectral profile. This approach demonstrated that SERS has the potential for direct detection and characterization of antibiotic resistance in real world samples instead of pure bacterial culture. Another study employed SERS-active AuNPs to study the antibiotic susceptibility of 12 urinary tract infection-causing bacteria [109]. Strain-specific identification was achieved with analytical sensitivity >95% and specificity >99%. The time for positive identification and AST was reduced to less than one hour.

In addition, SERS-active substrate can be employed as a means to establish MICs for various bacteria. Liu and colleagues demonstrated that SERS could monitor the reduction of specific bacterial biomarkers along with the treatment of antibiotics within two hours [110]. Clinical isolates of MRSA were exposed to vancomycin, while *E. coli*, *A. baumannii*, and *K. pneumoniae* were exposed to imipenem at the incremental concentrations. The isolates were determined as susceptible, intermediate, and resistant based on the change of the characteristic bands in SERS signals at a very early stage of antibiotic treatment, and the SERS MIC results were in excellent agreement with the standardized plate dilution methods that took upward of 24 h to complete. In a recent study, Cui and coauthors developed a homogeneous vacuum filtration-based method to improve SERS signal reproducibility and illustrated that the existence of heavy metal arsenic could increase the MIC of bacteria to the treatment of tetracycline. The authors claimed that SERS has the potential for culture-free characterization of resistome in a real microbiota system at the single cell sensitivity level [111].

Furthermore, monitoring the characteristic bacteria cell wall bands in the SERS spectra allowed for a further understanding of the antibiotic degradation mechanisms. The antibiotic response of *Lactococcus latis* was investigated using SERS-active AuNPs [112]. Antibiotic-induced spectral changes from ampicillin and ciprofloxacin were observed at 60 min after exposure to both antibiotics. However, ciprofloxacin induced only minor changes while ampicillin induced large SERS spectral changes. This was possibly because the inactivation mechanism of ciprofloxacin is to disrupt DNA synthesis, therefore the cell wall integrity was maintained for extended time periods and the cell wall signatures remained stable in the SERS spectra. While ampicillin interrupts the cell wall synthesis, which was directly detected by the SERS-active AuNPs. In another study, the SERS signals of *E. coli* were tracked upon antibiotic exposure to chloramphenicol, trimethoprim, polymyxin B, ampicillin, and formalin [113]. No spectral changes were observed after exposure to formalin although in vitro tests, which confirmed the cells were not viable. The authors noted that it was most likely due to the mechanism of formalin to crosslink membrane proteins but not degrade the cell wall. Similar results were observed with chloramphenicol and trimethoprim, which inactivate bacteria by inhibiting protein and DNA synthesis, respectively. The SERS signals remained unchanged after 2h exposure, which is possibly attributable to the sustained cell wall integrity. In contrast, SERS spectra changed within 5 min after antibiotic exposure to polymyxin B and ampicillin. They both aggressively degraded the bacterial cell wall, which released the SERS-active AgNPs and drastically reduced the SERS intensities. The technique could be used to further understand the fundamental mechanisms of microbial inactivation.

### 4.2. Chemometrics Used with SERS

Chemometric statistical analyses are usually required to decipher Raman spectral patterns so that minor variations in the spectral features of different biological samples can be distinguished. Multidimensional information of SERS spectra can be reduced into a few independent latent variables (called principal components) that account for the most variability of the original dataset by multivariate statistical analyses [114]. These principal components can then be used to segregate and quantify analytes based upon specific calibration models [115]. Chemometric methods include both unsupervised and supervised algorithms [116]. Among the spectroscopic-based pattern recognition methods, unsupervised principal component analysis (PCA) and hierarchical cluster analysis (HCA) are commonly used to provide either cluster plots or dendrogram structures for segregation and discrimination based upon the minor differences in Raman spectra [117]. Supervised chemometric models are generally used with some known answer from existing knowledge of the sample. Discriminant function analysis (DFA), partial least squares regression (PLSR), and soft independent modeling of class analog (SIMCA) are some of the most widely used models for the interpretation of SERS results [114]. For instance, a discriminant analysis is divided into two steps: to build a model using Raman spectra of bacterial cultures exposed to antibiotics of known class assignments, and to classify a new Raman spectrum of an antibiotic-exposed culture based on the distance to the multivariate mean of the closest class [118].

Different bacterial species or strains can be segregated into distinct groups based upon different biochemical compositions reflected by the major latent variables. For example, *E. coli*, *S. epidermidis* and four *Salmonella* strains exhibiting antibiotic resistance to the common therapeutics were detected and differentiated using SERS coupled with PCA [69]. In another work, SERS spectra of *P. mirabillis* and *Enterococcus* were quite similar despite having different cell wall structures. DFA was employed to analyze the subtle differences of SERS spectra from 6 strains of clinical urinary tract infection isolates for identification at genus-level [35]. Chemometric analysis play an important role in the determination of antibiotic resistance by SERS-based methods. Spectral differentiation of antibiotic resistant and sensitive strains can be demonstrated by chemometric models. For instance, Tien and others applied PCA for Raman spectra from MSSA and MRSA. MRSA cluster and MSSA cluster were segregated that can be used to differentiate MRSA from MSSA [119]. A SERS-based PLSR model was used to accurately determine the concentration of an MRSA strain in a mixture containing MSSA [54]. One recent study applied a three level chemometric model based on PLSR in combination with linear discriminant analysis (LDA) to extract those molecular changes and distinguish vancomycin-resistant and sensitive *Enterococci*. In addition, antibiotic-induced spectral changes from ampicillin and ciprofloxacin were monitored and statistically analyzed using PCA to understand the different working mechanisms of these antibiotics [112].

## 5. Conclusions and Future Direction

Raman spectroscopy and SERS have been validated for their potential in bacterial detection, typing, and characterization for almost three decades. Compared to the application of MALDI-TOF mass spectrometry for bacterial characterization, the use of Raman spectroscopy and SERS by industry is still in its infancy. This is mainly due to the relatively poor spectral reproducibility by using different types of the manufactured SERS substrates. As indicated in numerous review papers related to SERS bacterial study, to develop a stable SERS-active substrate for consistent and global use in a commercial manner is highly critical to promote this versatile technology to environmental, agri-food and clinical applications. Another major challenge is the relatively high cost of the confocal micro-Raman spectroscopic system. Although very little cost is required for purchasing consumables and instrumental maintenance compared to MALDI-TOF mass spectrometry, industries are still reluctant to purchase a bench-top Raman spectroscopic system. Therefore, a portable/handheld Raman instrument might be more affordable even though the resolution of the collected SERS spectra is relatively low. A more user-friendly software is also required for the convenient spectral interpretation as well as chemometric analyses. Several vendors have developed their own software for spectral processing and chemometrics, but a major doubt is how reliable such software for spectral analysis can be. By only clicking each “black-box” in the software, the researchers may not fully understand how each algorithm will affect the performance of the chemometric models. A standardized protocol for SERS spectral analyses and chemometric analyses therefore is critical to achieve inter-laboratory validation of the results for bacterial characterization, such as the characterization of bacterial antibiotic resistance.

Albeit these aforementioned challenges and potential limitations, SERS is definitely a very promising candidate for the determination of bacterial antibiotic resistance in a high-throughput, multiplex, and ultrafast manner. We suggest that industries use SERS for the detection and characterization of bacterial antibiotic resistance as an innovative fast screening alternative that can couple with the conventional methods for a further confirmation. Along with the further advancement in optical instrumentation and machine learning, the new version of the Raman spectroscopic system will be more user-friendly and cost-effective. We also envision that SERS can be used to further illustrate the modes of antibiotic and antimicrobial resistance of bacteria. This may contribute to the design of more effective antimicrobial treatment. Although SERS itself can be regarded as the core technology for an individual project, such as the detection of antibiotic resistance bacteria in a clinical specimen, we also believe it can be integrated as part of a more complicated study to drive very fundamental scientific research questions related to bacterial antibiotic resistance.

## Figures and Tables

**Table 1:** Summary of SERS-active nanomaterials used for the detection of bacteria.

SERS-Active Nanomaterial	Target Bacteria	LOD (CFU/mL)	LOQ (CFU/mL)	Sample Matrix	Detection Time	Chemometric Models	COMMENTS	Ref.
AgNPs	*E. coli*	8.0 × 10^2^	N/A	N/A	3.1 h	-	Direct, microfluidic	[51]
AgNPs	methicillin-resistant *S. aureus* (MRSA)	N/A	N/A	N/A	3.3 min	DFA, HCA	Direct, microfluidic concentration	[54]
AgNPs	*M. tuberculosis*	-	N/A	-	1 h	PCA, LDA	Direct, microfluidic concentration	[55]
AuNP surface	*K. pneumoniae*	N/A	N/A	N/A	30 min	PCA	Direct, fluoroquinolone-resistant	[56]
AgNPs	*E. coli*, *A. calcoaceticus*, *B. megaterium*, *P. aeruginosa*	N/A	N/A	N/A	N/A	N/A	Direct	[57]
AgNPs	*E. coli*, *S. cohnii*	N/A	N/A	N/A	10 s	N/A	Direct, convective assembly	[58]
AgNPs and AuNPs	*E. coli*, *S. cohnii*	N/A	N/A	N/A	N/A	N/A	Direct, layer-by-layer deposition	[45]
AgNPs	*E. coli*, *S. epidermidis*	2.5 × 10^2^	N/A	N/A	10 min	HCA	Direct, in situ adsorption	[46]
AgNPs	*E. coli, M. morganii*, *E. lactis, L. casei*	NA	N/A	N/A	<5 min	PCA	Direct, in situ synthesis	[59]
Ag nanospheres	*E. coli, S. typhimurium, S. aureus*	10	N/A	N/A	N/A	CVA	Direct, self-assembly, Ag nanocrystals	[60]
Ag nanorods	*A. baumannii*, *E. coli*, *K. pneumoniae*, *P. aeruginosa*, *S. aureus*	N/A	N/A	N/A	N/A	PCA, HCA, PLS-DA	Indirect, vancomycin-coated	[61]
Octupolar metastructures	*Brucella*	10^4^	N/A	N/A	N/A	N/A	Indirect, bacteriophage, EBL fabrication	-
Au nanorods	*E. coli*	3.5 × 10^1^	3.5 × 10^2^	N/A	<2 h	N/A	Indirect, Raman reporter, biotin-avidin, magnetic core	[62]
Ag nanocubes	*E. coli*	10^2^	N/A	N/A	-	N/A	Indirect, Raman reporter, polyclonal antibody	[63]
AgNPs, AuNPs, and Ag/Au core shell NP	*E. coli* O157:H7, *S.* Typhimurium, *S. aureus*	10^2^–10^3^	N/A	N/A	<30 min	N/A	Indirect, Raman reporter, aptamers, multiplex detection	[64]
Au “nanopopcorn” @ single wall carbon nanotubes	*E. coli*	10^2^	10^2^	N/A	-	N/A	Indirect, antibody, photothermal inactivation	[65]
AuNP @ graphene oxide	MRSA	5	N/A	N/A	-	N/A	Indirect, Raman reporter, photothermal inactivation	[66]
Au “nanoovals”	*E. coli*	2.1×10^2^	N/A	Chicken broth, apple juice, soil solution	50 s	N/A	Indirect, Raman reporter, antibody, DEP concentration	[67]
AuNPs	*Mycobacterium avium* subsp. *Paratuberculosis*	5.0 × 10^2^	N/A	Milk	<24 h	N/A	Indirect, Raman reporter, antibody	[68]
Au “nanopopcorn” @ graphene oxide	MRSA	10	N/A	N/A	-	N/A	Indirect, Raman reporter, aptamer	-
Ag nanorod arrays	*S.* Enteritidis, *S. enterica*	10^2^	N/A	Mung bean sprouts samples	-	PCA, PLS-DA	Indirect, vancomycin-coated surface	[69]
C	*S.* Typhimurium, *S. aureus*	10^3^	N/A	Spinach	N/A	N/A	Indirect, antibody, Fe_3_O_4_/SiO_2_ secondary NPs	[70]
Ag/SiO_2_ core/shell NPs	*S.* Typhimurium	10^8^	N/A	N/A	N/A	N/A	Indirect, Raman reporter, antibody	[71]
Au “nanopopcorn”	*S.* Typhimurium DT 104	10	N/A	Romaine lettuce	5 min	N/A	Indirect, Raman reporter, monoclonal antibody	[72]
SiO_2_/Au and Au/Ag core/shell NPs	*S.* Typhimurium	15	15	Milk	N/A	N/A	Indirect, Raman reporters, aptamers	[73]
Au/Ag core–shell nanoparticles	*V. parahaemolyticus*	10	10	N/A	N/A	N/A	Indirect, Raman reporters, aptamers	[74]
Au nanopopcorn	*S.* Typhimurium DT 104	10	N/A	N/A	N/A	N/A	Indirect, Raman reporter, antibody, photothermal inactivation	[75]
Fe_3_O_4_/Au core/shell NPs	*S.* Typhimurium, *S. aureus*	15	10^2^	Pork sample	N/A	N/A	Indirect, aptamer, magnetic separation	[76]
MnFe_2_O_4_/Au core/shell	*S. aureus*	10	N/A	Apple, pear, and grapes peels	N/A	N/A	Indirect, Raman reporter, aptamer, magnetic separation	[77]
Au nanoaggregate-embedded beads	*S. aureus*	N/A	N/A	N/A	N/A	N/A	Indirect, Raman reporter, antibody	[78]
AgNPs	*S. aureus*	15	15	Urine, blood, or pleural and ascites fluids	N/A	N/A	Direct, antibody, aptamer, Raman reporter	[79]
Fe_3_O_4_/Au core/shell NP	*S. aureus*	1	N/A	N/A	N/A	N/A	Indirect, antibody, magnetic concentration/separation	[80]
Au/Ag core/shell nanorod arrays	*S. xylosus*, *L. monocytogenes*, *E. faecium*	50	N/A	N/A	PCA	N/A	Indirect, Raman reporter	[81]
